# Book Reviews

**Published:** 1900-06

**Authors:** 


ULCER OF THE STOMACH AND DUODENUM AND ITS CONSEQUENCES. By Samuel Fenwick, M.D., F.R.C.P., Consulting Physician to the London Hospital, and W. Soltan Fenwick, M.D., M.R.C.P., London. Price $3.50. Philadelphia. P. Blakiston's Son & Co. 1900.

This extremely handsome volume of nearly 400 pages is devoted to an exhaustive consideration of ulcer of the stomach and duodenum and the complications and sequelæ arising from it. The work shows great care and study in preparation, and is written largely from personal experience of the authors, not to the neglect of the views and observations of others. It is well illustrated, and is the most comprehensive and valuable treatise upon the subject which has appeared in medical literature.

---

ESSENTIALS OF DIAGNOSIS. Saunders' Question Compends, No. 17. By Solomon Solis-Cohen, M.D., Philadelphia, and Augustus A. Eshner, M.D., Philadelphia. Illustrated. Second edition. Revised and enlarged. Price $1.00 net. Philadelphia. W. B. Saunders. 1890.

Saunders' Question Compends have proved of such value to students of medicine that they have become well-nigh indispensable. The present volume of the series deals with the essentials of diagnosis, and is a work of 400 pages, the subject-matter being arranged in the form of questions and answers. The text is freely illustrated. The authors are well-known writers and teachers, and the book will undoubtedly prove a valuable aid to the student in preparation for examination and in systematizing the course on practice.

---

A PRACTICAL TREATISE ON SEXUAL DISORDERS IN THE MALE AND FEMALE. By Robert W. Taylor, M.D., New York. Second edition. Thoroughly revised, with 91 illustrations and 13 plates in color and monochrome. Lea Bros. & Co., New York and Philadelphia.

The first edition of this work in a province of medicine in which the literature is not excessive served to introduce a new claim to the popularity of the well-known author. The second edition is an amplification of the first, the revision is paragraphic, and includes new chapters on masturbation in women, kraurosis vulvæ, vaginismus. The book is well illustrated. As a clear and entirely practical treatise on the subject it has no equal in English.

---

ESSENTIALS OF DISEASES OF THE SKIN, INCLUDING THE SYPHILODERMATA. By Henry W. Stelwagon, M.D., Ph.D. Philadelphia. Fourth edition. Revised. Illustrated. Price $1.00. Philadelphia. W. B. Saunders. 1899.

This condensed work on diseases of the skin has for some years enjoyed popularity. The subject is dealt with practically and concisely. Theoretical digressions and moot questions are avoided. The book is particularly full on treatment, and the illustrations are good—two points that will commend it to the student. The price puts it within the reach of all who desire a minor but reliable treatise on dermatology.

